# *In vitro* and *in vivo* antioxidant therapeutic evaluation of phytochemicals from different parts of *Dodonaea viscosa* Jacq

**DOI:** 10.3389/fchem.2023.1268949

**Published:** 2023-11-07

**Authors:** Siraj Khan, Mujeeb Ur Rehman, Muhammad Zafar Irshad Khan, Rehana Kousar, Khan Muhammad, Ihsan Ul Haq, Muhammad Ijaz Khan, Najla Almasoud, Taghrid S. Alomar, Abdur Rauf

**Affiliations:** ^1^ Department of Pharmacy, Faculty of Biological Sciences, Quaid-i-Azam University, Islamabad, Pakistan; ^2^ Cadson College of Pharmacy, Kharian, Pakistan; ^3^ Department of Pharmacy, Abasyn University Islamabad Campus, Islamabad, Pakistan; ^4^ Physiology Lab, Crop Sciences Institute, National Agricultural Research Centre (NARC), Islamabad, Pakistan; ^5^ Department of Pharmacy, University of Swabi, Swabi, Khyber Pakhtunkhwa, Pakistan; ^6^ Department of Chemistry, College of Science, Princess Nourah bint Abdulrahman University, Riyadh, Saudi Arabia; ^7^ Department of Chemistry, University of Swabi, Swabi, Khyber Pakhtunkhwa, Pakistan

**Keywords:** natural antioxidants, GSH, GST, catalase activity, hydrogen peroxide scavenging, free radical scavenging

## Abstract

**Introduction:** Natural antioxidants are vital to promote health and treat critical disease conditions in the modern healthcare system. This work adds to the index of natural medicines by exploring the antioxidant potential of *Dodonaea viscosa* Jacq. (Plant-DV).

**Material and Methods:** The aqueous extract of leaves and flower-containing seeds from plant-DV in freshly prepared phosphate buffer is evaluated for antioxidant potential. *In vitro* antioxidant potential of the nascent and oxidatively stressed extracts was analyzed through glutathione (GSH) assay, hydrogen peroxide (H_2_O_2_) scavenging effect, glutathione-S-transferase (GST) assay, and catalase (CAT) activity. *In vivo* therapeutic assessment is performed in Wistar Albino rats using vitamin C as a positive control. The livers and kidneys of individual animals are probed for glutathione, glutathione-S-transferase, and catalase activities.

**Results:** flower-containing seeds have GSH contents (59.61 µM) and leaves (32.87 µM) in the fresh aqueous extracts. The hydrogen peroxide scavenging effect of leaves is superior to flower-containing seeds with 17.25% and 14.18% respectively after 30 min incubation. However, oxidatively stressed extracts with Ag(I) and Hg(II) show declining GSH and GST levels. The plant extracts are non-toxic in rats at 5000 mg/Kg body weight. Liver and kidneys homogenate reveal an increase in GSH, GST, and CAT levels after treatment with 150 ± 2 mg/kg and 300 ± 2 mg/kg body weight plant extract compared with normal saline-treated negative and vitamin C treated positive control.

**Discussion:** The crude aqueous extracts of leaves and flower-containing seeds of plant-DV show promising antioxidant potential both in *in vitro* and *in vivo* evaluation.

## 1 Introduction

A profound biological response of specific chemical entities in natural extracts opens new horizons to novel medicines. Natural medicines are derivatives of plants, animals, microbes, marine, and other natural sources that play an important role in the modern healthcare system (Sidra et al., 2014). Some biological molecules exhibit important antioxidant functions, adding to the immune power of plants and animals. Antioxidants protect the cell from oxidative damage by either scavenging or repairing damaged molecules. The intake of enough antioxidants is supposed to protect the body against certain diseases (Jayachitra and Krithiga, 2012; Subramanian et al., 2023) like neurodegeneration, aging, viral infections, and cancer (Kamran et al., 2020).

Evaluation of glutathione-*S*-transferases (GSTs) is a potential screening method to explore the antioxidant strength of the extract. Its rich level is expected to offer a diverse defense system to living cells. GST is involved in the inactivation of xenobiotics, and it scavenges toxic substances and prevents the formation of free radicals (Aras et al., 2023). Hydrogen peroxide (H_2_O_2_) is the most abundantly produced endogenous free radical and is widely known to produce oxidative stress. The elevated level of H_2_O_2_ is associated with suboptimal health status and needs to be warranted in any living system. Catalase protects the cell from oxidative damage by converting hydrogen peroxide to water and molecular oxygen (Dziechciarz et al., 2023). The cellular environment requires optimum glutathione (GSH) stores to protect itself from damage caused by a number of oxidative species. Glutathione is a naturally occurring low-molecular weight non-protein thiol molecule. The presence of the sulfhydryl (SH) group has made it uniquely important by conferring antioxidant potential. It protects the cells from free radicals and other similar oxidizing species (Jones, 2002).

Heavy metals are widespread pollutants of greater concern as they are non-degradable and, thus, persistent threats of oxidative toxicities. They are one of the main environmental pollutants as they remain in the environment for longer periods. Their accumulation is extremely hazardous to life including plants, animals, and humans (Benavides et al., 2005). A quantitative study about antioxidant biomarkers concerning silver and mercury salts would help interpret data from situations of much more complex metal exposure. Additionally, the interaction studies would present more realistic insights into the exposure of plants to these metal mixtures in the environment. The study of the effects of Ag(I) and Hg(II) salts on small thiols and proteins provides a good model to establish this process of conjugation at the molecular level.

The genus *Dodonaea* (Sapindaceae)—a shrub—is geographically distributed in the temperate regions of Australia, South America, Mexico, Africa, India, and Florida (Mostafa et al., 2014). *Dodonaea* is one among 140 genera and consists of approximately 68 species (Simpson et al., 2011). Traditionally, *Dodonaea viscosa* Jacq. is used in the treatment of various ailments such as rheumatoid arthritis, diarrhea, stomach pain, skin infections, hepatic or splenic pain, uterine cramp, and other ailments involving dermatitis, smooth muscles, hemorrhoids, and sore throat (Al-Snafi, 2017).

*Dodonaea viscosa* Jacq. (plant DV) has antioxidant (Alanazi et al., 2023), antidiabetic (Malik et al., 2022), anti-inflammatory (Siddiqui et al., 2023), anticholinesterase (Muhammad et al., 2016), and cytotoxic activities (Herrera-Calderon et al., 2023), but detailed screening of the enzymatic and non-enzymatic antioxidant phytochemicals remains to be explored.

A previous study reported quercetin and isorhamnetin in the root bark and kaempferol in its leaf extract (Getie et al., 2003). Diterpenes and nor-diterpenes are also reported in *D. viscosa* leaves (Marvilliers et al., 2020). This study undertakes the most vital assays, including the GSH assay, H_2_O_2_ scavenging effect, GST assay, and catalase (CAT) activity, to screen the antioxidant worth/value of *D. viscosa* Jacq. (Al-Snafi, 2017).

## 2 Materials and methods

### 2.1 Chemicals

The chemicals used include GSH (FLUKA), 5, 5-dithio-bis (2-nitrobenzoic acid) (DTNB) (Sigma-Aldrich), 1-chloro-2,4-dinitrobenzene (CDNB) (Sigma-Aldrich), hydrogen chloride (Kolchlight), ascorbic acid (Merck), mercury(II) acetate [Hg(OAc)₂] (Sigma-Aldrich), and silver nitrate (AgNO_3_) (Sigma-Aldrich).

### 2.2 Equipment

The equipment used includes a UV-Vis double-beam spectrophotometer, HALO DB-20 (Dynamica, Australia), centrifuge (Hermle Labortechnik Germany), hot plate 400 device (England), sonicator (SweepZone Technology, United States), and Freezer and Rotary Evaporator RE200 (Bibby Sterilin Ltd., England).

### 2.3 Preparation of plant material and extraction

#### 2.3.1 Collection and identification

Fresh flower-containing seed and leaves of the plant were collected during the flowering season from Quaid-i-Azam University, Islamabad, Pakistan. The plant was identified in the Department of Plant Sciences, Quaid-i-Azam University, Islamabad, and a voucher specimen (PHM-499) was deposited in the herbarium of medicinal plants.

#### 2.3.2 Drying

The collected plant material was sorted for any unwanted herbs and decayed or rotten plant parts. The sorted parts were thoroughly rinsed with tap water. The leaves and flower-containing seed were collected separately and air-dried at room temperature in shade for up to 3 weeks until easy crumbling. Flower-containing seeds were collected, and the thin membrane was removed by tumbling and aeration. Appropriately dried plant materials were then pulverized into coarsely grounded powder. The powder was individually filled in polythene jars and tightly sealed until further use.

#### 2.3.3 Extraction

The collected powder of leaves and flower-containing seeds was macerated in phosphate buffer for 2 h. The homogenate was stirred and filtered through a muslin cloth. The filtrate was centrifuged at room temperature at 2,000 × *g* for 10 min to remove any undissolved material. Supernatants were collected in well-closed Falcon tubes, freeze-dried, and stored at −20°C until further use.

### 2.4 *In vitro* assessment of antioxidant potential

#### 2.4.1 Estimation of reduced GSH

**FIGURE 1:**
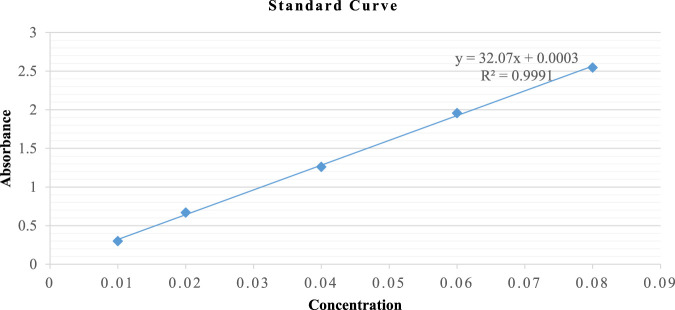
Standard curve of GSH.

Reduced glutathione was determined by GSH assay (Moron et al., 1979) with slight modifications, as shown in Figure 1. Powder weighing 1 g/5 mL of each portion was macerated in 5% TCA solution (phosphate buffer 0.2M, pH 8.0) and continuously stirred using a magnetic stirrer for 120 min. Subsequently, the extract was centrifuged at RCF = 67 × *g* for 10 min, and the supernatant was collected. The flower-containing seeds supernatant was serially diluted with phosphate buffer (0.2M, pH 8.0) into five different concentrations at extract-to-phosphate buffer ratios (0.2M, pH 8.0) of 7:0, 6:1, 5:2, 4:3, and 3:4. The leaves supernatant was serially diluted with phosphate buffer (0.2M, pH 8.0) into five different concentrations at extract-to-phosphate buffer ratios of 1:5, 1:6, 1:7, 1:8, and 1:9. Normal levels of GSH in each of the five samples were determined spectrophotometrically.

DTNB (2 mL) and phosphate buffer (1 mL) were used as control. The samples were prepared by mixing DTNB (0.6 mM in 0.2 M phosphate buffer, 2 mL), buffer (0.9 mL), and respective concentrations of the supernatant (0.1 mL). These mixtures were incubated for 10 min at room temperature under dark conditions. The absorbance of the assay mixture was spectrophotometrically measured at *λ* = 412 nm in triplicate, using phosphate buffer as a blank. The values were expressed as µM GSH/g of powder using a standard GSH calibration curve.

#### 2.4.2 Oxidative stress with metals

Serially diluted five different concentrations were prepared for each metal salt, that is, mercury(II) acetate [Hg(OAc)₂], and silver nitrate (AgNO_3_). Stock solutions of the metal salts were prepared to be stoichiometrically equivalent to the highest concentration of the respective plant extract. The concentration of the respective plant extract was calculated from UV-Vis spectrophotometric absorbance and presented in molar units. The metal salt (0.1 mL) was added to the assay mixture of different dilutions and measured spectrophotometrically after incubation of 10 min at wavelength *λ* = 412 nm using phosphate buffer (0.2 M, pH 8.0) as a blank.

#### 2.4.3 Scavenging of hydrogen peroxide

The scavenging effect of extracts on H_2_O_2_ was determined over an extended period by a method reported by Ruch et al. (1989). The leaves and flower-containing seed powder samples were extracted separately, as per the method previously described. The H_2_O_2_ scavenging effect was determined concerning different time intervals. The scavenging effect for each time interval was assessed spectrophotometrically at *λ* = 230 nm using phosphate buffer (0.1 M, pH 7.4) as a blank. The samples were analyzed and compared with the control (ascorbic acid).

Briefly, 10 µL of the plant supernatant was added to the H_2_O_2_ solution (40 mM; 0.6 mL), and the total volume was adjusted to 3 mL. The absorbance was recorded at *λ* = 230 nm using a spectrophotometer. The H_2_O_2_ scavenging value of the plant extracts was calculated as

$$\%\;H_{2}O_{2}\text{ scavenging}=\frac{\left(A^{0}\;–\;A^{1}\right)\times 100)}{A^{0}},$$

where A^0^—absorbance of the control; A^1^—absorbance in the presence of the plant extract.

#### 2.4.4 Glutathione-S-transferase estimation

Glutathione-*S*-transferase was assessed by GST assay, as reported by Habig et al. (1974). The basic principle of the assay is the ability of GST to catalyze GSH and CDNB conjugation. The activity of the enzyme was determined by observing the change in absorbance at *λ* = 340 nm. The samples were prepared by homogenizing 2 g of relevant plant-DV powder in 20 mL phosphate buffer (0.1 M, pH 6.5). Homogenates were centrifuged at RCF = 1,680 × *g* for 10 min, and the supernatant was collected. Different serial dilutions were prepared by adding phosphate buffer (0.1 M, pH 6.5) to the extract. The supernatant of flower-containing seeds was serially diluted with phosphate buffer into five different concentrations at extract-to-phosphate buffer ratios of 7:0, 6:1, 5:2, 4:3, and 3:4. The leaves extract was serially diluted with phosphate buffer (0.1 M, pH 6.5) into five different dilutions at extract-to-phosphate buffer ratios of 1:5, 1:6, 1:7, 1:8, and 1:9. The reaction mixture contained GSH (0.1 mL), CDNB (0.1 mL), and phosphate buffer (0.1 M, pH 6.5) in a total volume of 2.9 mL. The reaction was initiated by adding the enzyme extract (0.1 mL). The absorbance readings were recorded at *λ* = 340 nm using a UV-Vis spectrophotometer. The assay mixture without the extract served as a control to monitor the nonspecific binding of the substrates. GST activity was calculated using the extinction coefficient (9.6 mM^−1^cm^−1^) of the product formed and was expressed as µM of conjugated CDNB (µmol/min/mg).

#### 2.4.5 Oxidative stress of the extract with metals

Five different dilutions were prepared for each metal salt mercury(II) acetate Hg(OAc)₂ and silver nitrate AgNO_3_. Stock solutions of the metal salts were prepared to be stoichiometrically equivalent to the highest concentration of the respective plant extract. The concentration of the respective plant extract was calculated from UV-Vis spectrophotometric absorbance and presented in molar units. The serial dilutions of the supernatant and metals were mixed, shaken, and incubated for 10 min. After incubation, CDNB (0.1 mL), the reaction mixture (supernatant and metal, 0.1 mL), GSH (0.1 mL), and 2.7 mL phosphate buffer (0.1 M, pH 6.5) were added to adjust the volume of 3 mL, and the absorbance was recorded at *λ* = 340 nm using phosphate buffer (0.1 M, pH 6.5) as a blank. The assay mixture without the extract served as a negative control to monitor the nonspecific binding of the substrates. The absorbance of the positive control was also recorded. All readings were taken in triplicate. GST activity was calculated using the extinction coefficient (9.6 mM^−1^cm^−1^) of the product formed and was expressed as µMol of CDNB conjugated/min (µmol/min/mg).

#### 2.4.6 Assessment of catalase activity

Catalase activity was determined by the catalase assay previously reported (Naz et al., 2022). An amount of 4 g of relevant plant-DV powder was mixed in phosphate buffer (0.067 M, 20 mL, pH 7.0) through magnetic stirring. Subsequently, the extract was centrifuged at RCF = 67 × *g* for 15 min at 4°C, and the supernatant was collected. The supernatant was serially diluted with phosphate buffer into five different concentrations. Catalase activity in each diluted sample was assessed spectrophotometrically at *λ* = 240 nm. Samples were assessed against absorbance readings of the positive control. The phosphate buffer (0.067 M, pH 7.0) and supernatant were considered the blank for each sample and served as a negative control. The H_2_O_2_–phosphate buffer (2 mM) was used as a positive control. In the experimental cuvette, 40 μL of the enzyme extract was added to the H_2_O_2_–phosphate buffer (3 mL) mixture and mixed thoroughly. The decrease in absorbance by 0.05 units was noted at *λ* = 240 nm and was expressed as unit/mg protein.

The amount of enzyme unit was calculated as the decrease in absorbance at *λ* = 240 nm by 0.05 units.

### 2.5 *In vivo* assessment of the antioxidant effect

#### 2.5.1 Animals

Wistar albino rats (weight 150 g and 200 g) were used in the current study to evaluate the *in vivo* antioxidant activity. All experimental animals were purchased from the National Institute of Health (NIH), Islamabad, Pakistan. These animal studies were reviewed and approved by the ethical committee of Quaid-i-Azam University, Islamabad, under the assigned protocol number BEC-FBS-QAU2017-179. Antioxidant activities were performed at a constant temperature of 25°C ± 2°C in a pathogen-free zone of the Department of Pharmacy, Quaid-i-Azam University, Islamabad (Pakistan), as per the standard procedure for the care and use of laboratory animals (Quaid-i-Azam University). The animals were housed in plastic cages (six rats per cage) having free contact with food and water. All animals *n* = 48 were categorized into four groups, comprising control and treated groups. Group 1, negative control, was treated with normal saline, group 2, positive control, was treated with 50 ± 2 mg/kg ascorbic acid, and groups 3 and 4 were each treated with 150 ± 2 mg/kg and 300 ± 2 mg/kg plant extracts, respectively. Each dose of the extract was dissolved in 1 mL distilled water and administered orally by oral gavage. Carbon tetrachloride (CCl_4_) 0.5 mL/kg body weight and 20% CCl_4_/olive oil were injected intraperitoneally into each rat twice a week for 8 weeks. After 24 h from the last dose, all animals were anesthetized with chloroform and euthanized by cervical dislocation. The liver and kidneys from each animal were collected, washed with ice-cold saline, patted dry, and weighed. The extracted tissues were individually homogenized in Tris HCl buffer (pH 7.4) to prepare the homogenate. Each tissue homogenate was centrifuged at RCF = 1,075 × *g* at 4°C for 15 min. The supernatant was collected and used for the estimation of reduced GSH, GST, and CAT.

#### 2.5.2 Acute toxicity test (LD_50_)

The acute toxicity test (LD_50_) was performed, as previously reported (Khalil et al., 2006). The test extract was administered at different doses (100 mg/kg–5,000 mg/kg) to different groups. Immediately after dosing, the animals were observed every 4 h for 4 days to determine mortality.

#### 2.5.3 Determination of reduced GSH

Reduced glutathione was quantified by the GSH assay, as reported in Ellman (1959). The assay is based on the oxidation of GSH by 5,5-dithiobis (2-nitrobenzoic acid) (DTNB). DTNB and GSH react together, producing 2-nitro-5- thiobenzoic acid (TNB) having a yellow color. Briefly, the sample was prepared by mixing DTNB (2.4 mL), buffer (0.5 mL), and respective dilution of the supernatant (0.1 mL). The GSH concentration was determined by measuring absorbance at 412 nm using a UV-Vis spectrophotometer using phosphate buffer as a blank.

#### 2.5.4 Determination of GST

The GST concentration was determined by the GST assay, as previously described (Farombi et al., 2007; Doherty et al., 2010). The GST enzyme is capable of conjugating GSH and CDNB. Concisely, the GST level in liver and kidney tissues was estimated by mixing 0.1 mL of the supernatant from the respective homogenate and 0.1 mL of CDNB. Finally, 0.1 M phosphate buffer (pH 6.5) was added to obtain a final volume of 3 mL. The reading was observed using the UV-Vis spectrophotometer at *λ* = 314 nm. Absorbance was measured in triplicate using a UV-Vis spectrophotometer at *λ* = 314 nm using distilled water as a blank.

The animals were randomly divided into two groups (*n* = 8/group). CCl_4_ (0.5 mL/kg 20% CCl_4_/olive oil) was administered intraperitoneally (i.p.) twice a week for 8 weeks. At the same time, each animal was individually administered the plant-DV extract (150 ± 2 mg/kg and 300 ± 2 mg/kg b.w.) in distilled water orally, twice a week for 8 weeks. At the end of week 8, 24 h after the last treatment, animals were euthanized, as mentioned previously. The kidneys and liver were removed and washed in ice-cold normal saline and cryopreserved in liquid nitrogen and stored at −80°C.

#### 2.5.5 Determination of catalase activity

The method previously reported (Naz et al., 2022) was used to determine catalase activity. Phosphate buffer (0.1 M, pH 7.4) was taken as a blank. The samples were analyzed against a control. A measure of 40 µL of the plant supernatant was added to the H_2_O_2_ solution (40 mM; 0.6 mL), and the total volume was made up to 3 mL and mixed thoroughly. The final reading was noted at 240 nm in triplicate.

### 2.6 Statistical analysis

The statistical analysis was carried out using OriginPro 8.5 and Microsoft Excel 2019. All the processes were performed in triplicate, and the results were expressed (*n* = 3) as mean ± standard deviation (SD).

## 3 Results and discussion

### 3.1 Assessment of GSH levels

#### 3.1.1 Flower-containing seed exhibits higher GSH potential than leaves in *in vitro* assay

**FIGURE 2:**
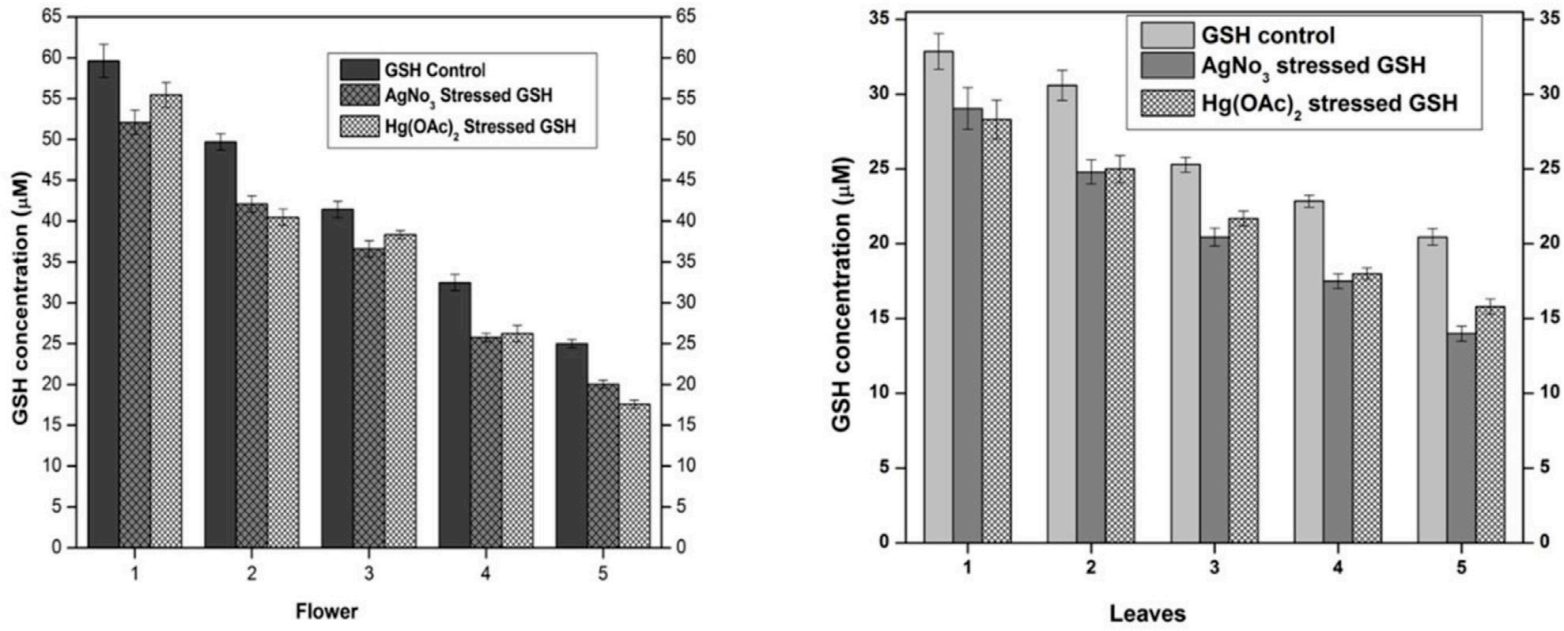
Normal and stressed Ag(I) and Hg(II) levels of GSH expressed in µM, in both flower and leaf parts of plant DV. The concentration-dependent effect in five serial dilutions is determined. Values are expressed as mean ± SD (*n* = 3); *p* < 0.05.

The supernatant of flower-containing seeds and the leaf part of plant DV was evaluated for normal GSH content without the influence of any exogenous or endogenous factor. The normal GSH concentration in seeds and leaves parts of the plant DV termed control is shown in Figure 2. GSH levels were calculated and presented in molar units obtained from the standard curve. The undiluted sample of flower-containing seeds and the leaves supernatant was estimated to be 59.61 and 32.87 µM of GSH, respectively. The GSH concentration was noted to be 24.97 and 20.45 µM in the flower-containing seed part and leaves, respectively, as the supernatant was diluted with aqueous phosphate buffer. After the determination of normal GSH levels, the relevant extracts were subjected to oxidative stress by exogenous silver nitrate and mercury(II) acetate. The strength of the Ag(I) stock solution for the flower-containing seeds and leaves was selected to be 58.34 and 35 µM, respectively, to keep this metal solution stoichiometrically equivalent to the GSH concentration. The strength of the Hg(II) stock solution for the flower-containing seeds and leaves was selected to be 29.17 and 17.5 µM, respectively, to keep the metallic solution stoichiometrically half to the GSH concentration in undiluted extracts. The spectroscopic evaluation of the GSH concentration showed a decrease in undiluted and diluted flower-containing seed and leaves supernatants. These metals [silver nitrate and mercury(II) acetate] showed depletion in each dilution of plant DV. The GSH concentration in the flower-containing seeds supernatant decreased from 59.61 µM to 52.08 µM and 55.56 µM with Ag(I) and Hg(II)-treated metals, respectively, in the undiluted supernatant. The GSH concentration in the leaf supernatant also decreased from 32.87 µM to 29.05 µM and 28.3 µM with Ag(I)- and Hg(II)-treated metals, respectively, in the undiluted supernatant. The GSH concentration also decreased with dilution in both flower-containing seeds and leaves supernatant, as shown in Figure 2. Metals such as Ag(I) and Hg(II) induced stress to reduce the GSH concentration by binding to the sulfhydryl group of GSH. The idea was based on the previous reports (Al-Snafi, 2017). GSH contains a thiol group with a metal scavenging affinity. A previous study showed that heavy metal toxicity increases with increasing concentration, resulting in decreased GSH levels (Jozefczak et al., 2012).

The primary mechanism of heavy metal toxicity in humans is the formation of organometallic complexes that block other biological pathways. When heavy metals interact with the sulfhydryl groups in the non-enzymatic antioxidant system (e.g., replacing a hydrogen atom on the reduced GSH moieties), these complexes are formed (Srnovršnik et al., 2023). Additionally, the reduced GSH content due to heavy metal treatment favors the production of reactive oxygen species (Gallego et al., 1996).

#### 3.1.2 Flower-containing seeds and leaves extract of plant DV enhanced GSH content in the liver and kidneys of treated animals

**FIGURE 3:**
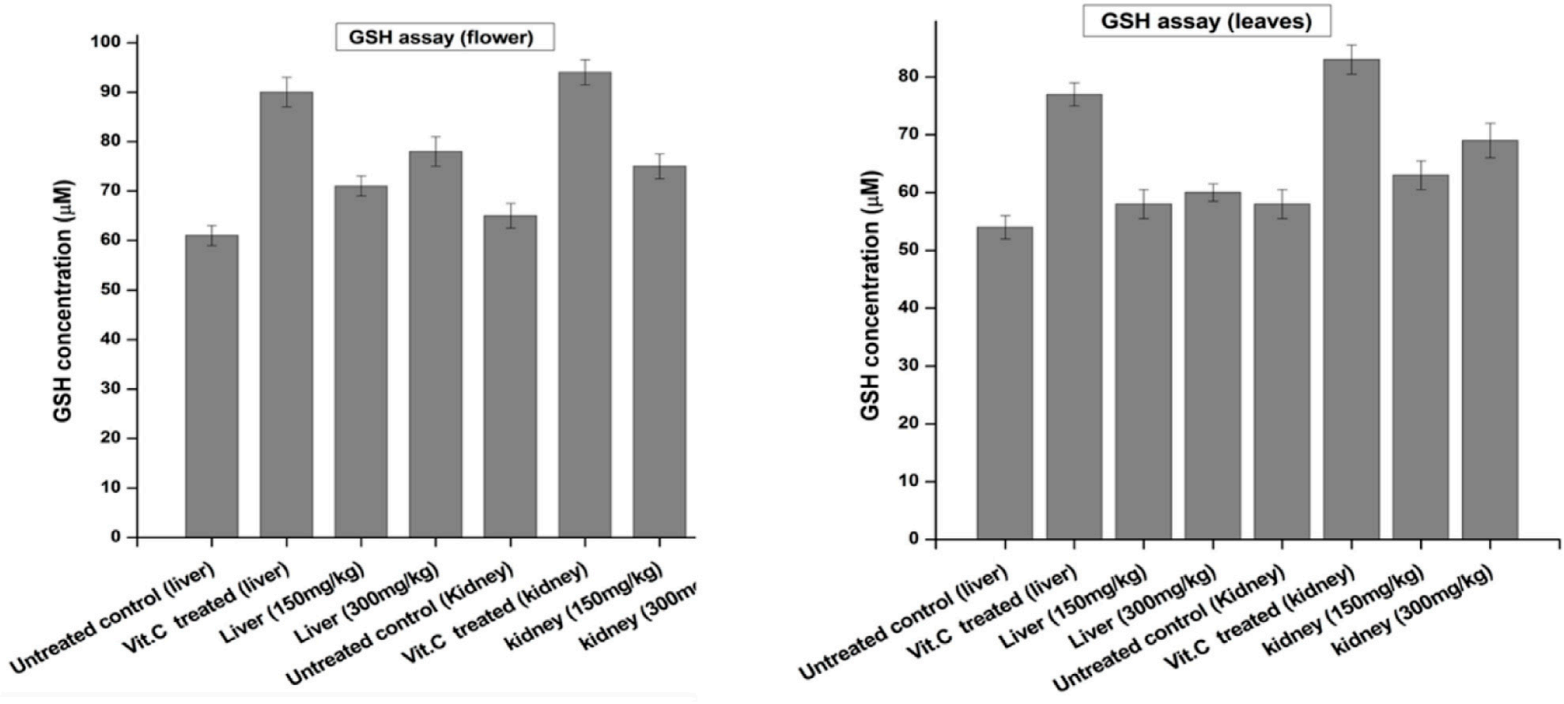
Levels of reduced GSH in the liver and kidney of control and experimental groups of rats. Data were given as mean ± standard deviation for animals in each group; *p* < 0.05.

The GSH concentration was determined in the liver and kidneys of euthanized animals and compared with the negative control and standard as a positive control. Vitamin C was used as a standard drug for its antioxidant effect. Each group was assayed by the method previously reported by Moron et al. (1979). As shown in Figure 3, the GSH concentration of the flower-containing seed part of plant DV was found to be 71 μM at the dose of 150 ± 2 mg/kg and 78 μM at the dose of 300 ± 2 mg/kg in the liver. However, the negative control group yielded 61 µM, and the standard group/positive control treated with vitamin C showed 90 µM. In the kidney tissue, the GSH concentration of the flower-containing seed extract-treated group was found to be 75 μM at the dose of 150 ± 2 mg/kg and 82 μM at the dose of 300 ± 2 mg/kg compared to the concentration of 65 µM of the negative control group and 94 µM of the vitamin C-treated standard group. The obtained results of leaf extract-treated animals indicated that the doses of 150 ± 2 mg/kg and 300 ± 2 mg/kg increased the GSH concentration in liver tissue at 58 and 60 µM, respectively, compared with the concentrations of 54 µM and 77 µM of the negative control group and vitamin C-treated group, respectively. In the kidney tissue, the concentration of GSH showed an increase of 63 μM at the dose of 150 ± 2 mg/kg and 69 μM at the dose of 300 ± 2 mg/kg, compared to the concentrations of the negative control group, 58 µM, and vitamin C-treated group, 83 µM.

Glutathione is one of the most abundant tripeptides present in the liver, functioning as an enzymatic antioxidant. It is primarily concerned with the removal of free radical species such as hydrogen peroxide, superoxide radicals, and alkoxy radicals. It also maintains the membrane protein thiol and works as a substrate for glutathione peroxidase and GST (Prakash et al., 2001; Zhu et al., 2023). Our results showed that the extract caused an increase in GSH levels compared to the negative control group. GSH is one of the most vital antioxidant molecules exerting its antioxidant activity either through thiol conjugation or as an electron donor from its sulfhydryl group (Skenderidis et al., 2018).

### 3.2 Assessment of the hydrogen peroxide scavenging effect

#### 3.2.1 The leaves part of plant DV shows better scavenging of hydrogen peroxide than flower-containing seeds

**FIGURE 4:**
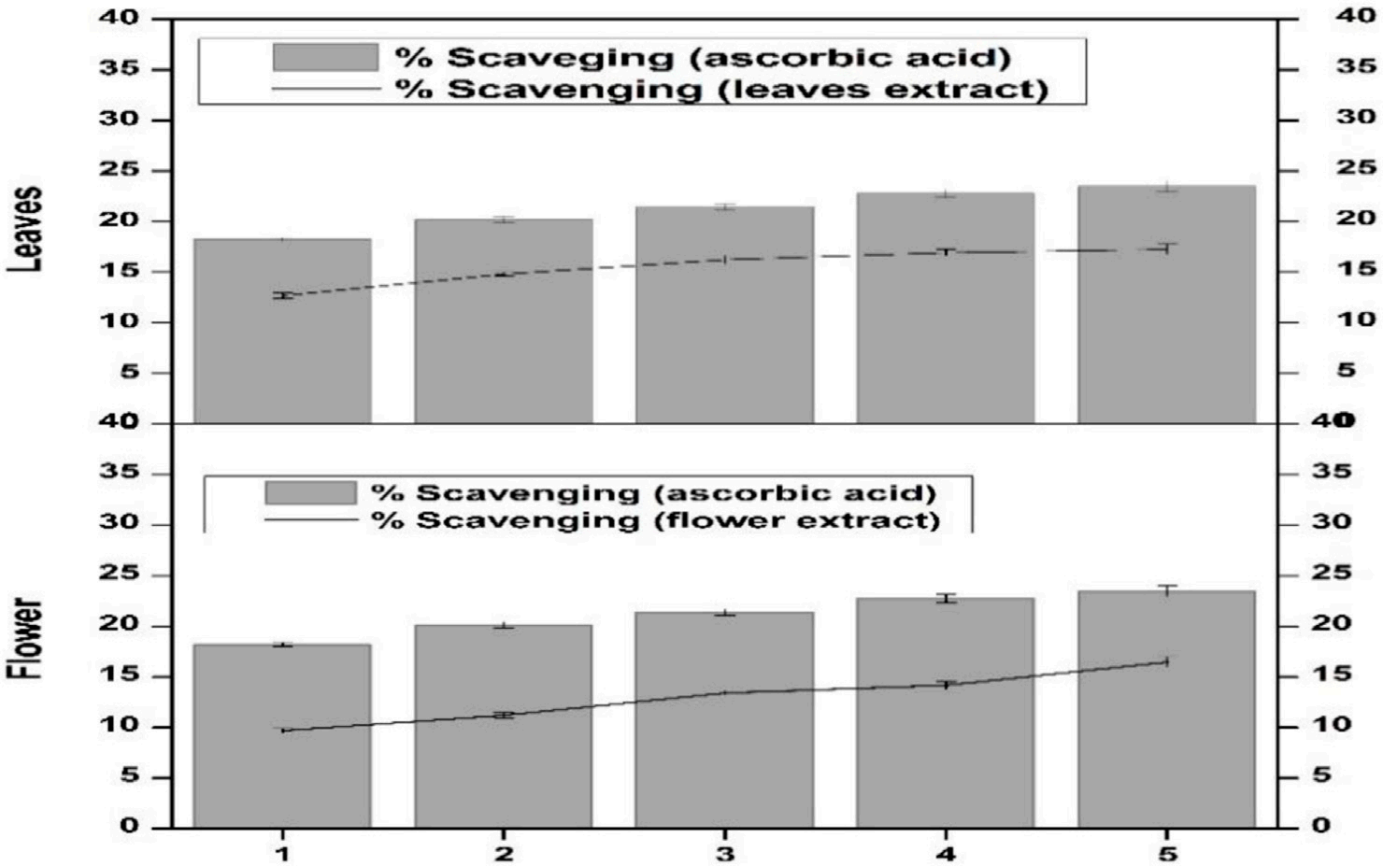
Hydrogen peroxide scavenging effect of the flower-containing seed and leaf extract in plant DV. Values are expressed as mean ± SD (*n* = 3); *p* < 0.05.

Scavenging activity of the flower-containing seeds and leaf part of plant DV was assessed for determining the H_2_O_2_ scavenging effect. The percentage scavenging effect was calculated according to the protocol reported by Ruch et al. (1989). The percentage scavenging effect was time-dependent and continuously increased as an effect of incubation time. After 1 min incubation, the scavenging effect was low in both plant parts (Figure 4). After 5 min incubation, the flower-containing seeds and leaf part showed a 9.7% and 12.67% scavenging effect, respectively. However, after 30 min incubation, the scavenging effect of the flower-containing seeds increased to 14.18% and that of leaves increased to 17.25%. Vitamin C was kept as a standard or positive control. The leaf extract showed a greater percentage scavenging effect than flower-containing seeds showing closer results to vitamin C. The scavenging effect seems to be due to catalase, peroxidase, and the presence of flavonoids in plant DV (Shalaby et al., 2012).

### 3.3 Assessment of GST levels

#### 3.3.1 Oxidative stress reduced the GST levels in a concentration-dependent manner

The supernatant of flower-containing seeds and leaves of plant DV was evaluated for normal GST levels in serially diluted samples. GST levels were presented in molar units (µmol/min/mg) converted from the spectrophotometric absorbance using the extinction coefficient (9.6 mM^−1^cm^−1^) of the product formed.

$$\text{GSH}+\text{CDNB}---------->\underset{\left(\text{Product}\right)}{\text{ GS}.\text{DNB}}+H^{+}+{\text{Cl}}^{-}$$

The reaction of GSH and CDNB is catalyzed by the plant enzyme GST to form the product GS-DNB having absorbance in the visible region at *λ* = 340 nm. The absorbance values obtained by GS-DNB product formation are directly related to the GST potential of each supernatant dilution of plant DV. The extinction coefficient (9.6 mM^−1^cm^−1^) used to convert the spectrophotometric absorbance values into molar units (µmol/min/mg) belongs to the GS-DNB product. Normal levels of GST in each of the five dilutions were assayed spectrophotometrically by GST assay (Habig et al., 1974).

**FIGURE 5:**
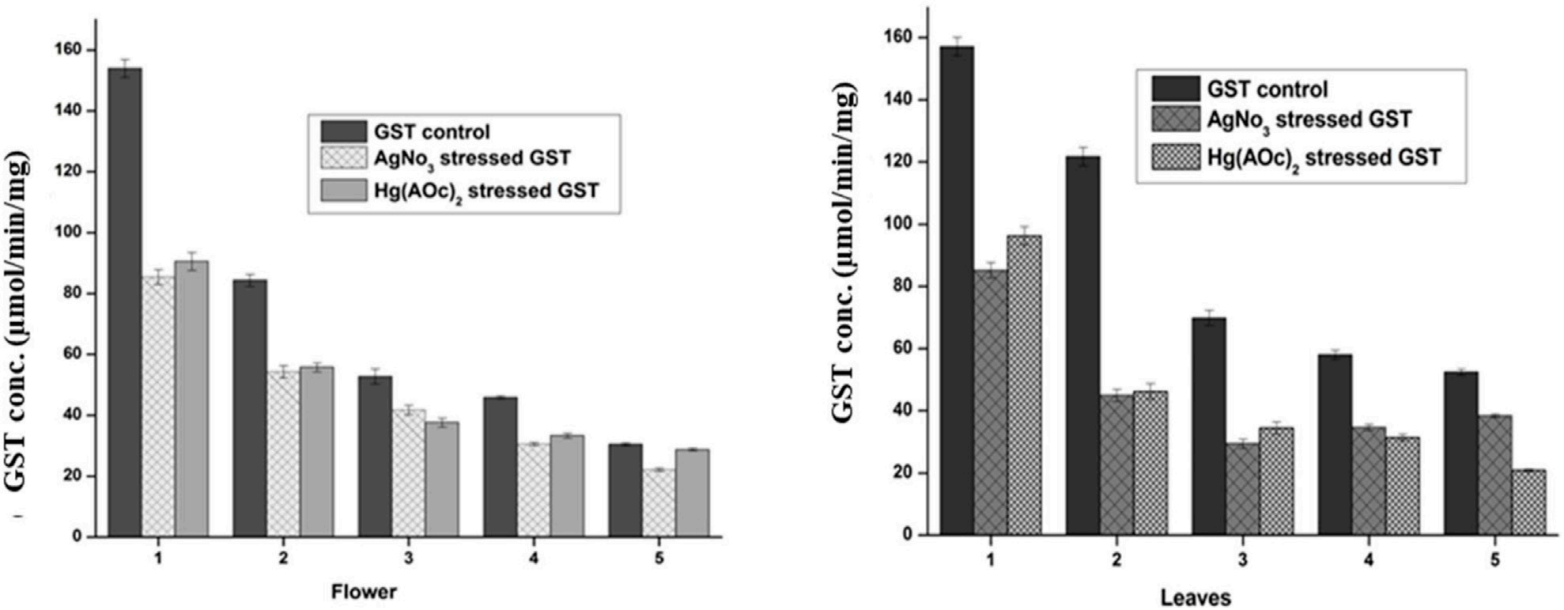
Normal and stressed Ag(I) and Hg(II) levels of GST expressed in µM, in both the flower-containing seed and leaf part of plant DV. Concentration-dependent effects in five serial dilutions are determined. Values are expressed as mean ± SD (*n* = 3); *p* < 0.05.

The GST level in the flower-containing seed extract was found to be higher (153.92 μmol/min/mg) in the maximum supernatant concentration (DV-1) and lowest (30.41 μmol/min/mg) in the lowest supernatant concentration (DV-5). Similarly, the GST level in the leaves was found to be higher (157.08 μmol/min/mg) in the sample having a maximum supernatant concentration (DV-1) and lowest (53.36 μmol/min/mg) in the sample having a minimum supernatant concentration (DV-5), as shown in Figure 5. These findings have a positive correlation with previous studies where plant DV was reported to hold significant antioxidant potential (Samavati and Manoochehrizade, 2013). The metal salt of Ag(I) as an exogenous oxidizing agent was incubated with the supernatant in the same stoichiometric ratio to each serially diluted sample. The GST level decrease was observed with the treatment of similarly diluted samples of Ag(I) in the flower-containing seeds of plant DV (Figure 5). The GST level decreased from 153.92 to 85.42 μmol/min/mg and from 30.41 to 22.11 μmol/min/mg in the most concentrated and most diluted samples, respectively. The GST level in the leaves part decreased in a consistent and concentration-dependent manner with the treatment of each dilution of Ag(I). The GST level in more concentrated samples decreased from 157.08 to 85.17 μmol/min/mg and in diluted samples from 52.36 to 38.40 μmol/min/mg. Treatment with Hg(II) also showed a concentration-dependent decrease in GST content. Every dilution of Hg(II) decreased the GST status of plant DV, as shown in Figure 5. The GST level of the flower-containing seed extract decreased from 153.92 to 72.92 μmol/min/mg and from 30.41 to 28.75 μmol/min/mg in the in highly concentrated and most diluted samples, respectively. The GST level in more concentrated samples of the leaves part decreased from 157.08 to 96.08 μmol/min/mg and from 52.36 to 20.84 μmol/min/mg in diluted samples.

Heavy metals may inactivate enzymes by binding to a cysteine residue; thus, the enzyme concentration decreases. The metal binds to sulfhydryl groups of enzymes and proteins, leading to misfolding and inhibition activity (Hossain et al., 2012). These metals might form a conjugate with GST, consequently leaving lesser GST available to catalyze the reactants, i.e., GSH and CDNB.

#### 3.3.2 Leaves showed better GST activity than the flower-containing seed extract of plant DV in *in vivo* evaluation

**FIGURE 6:**
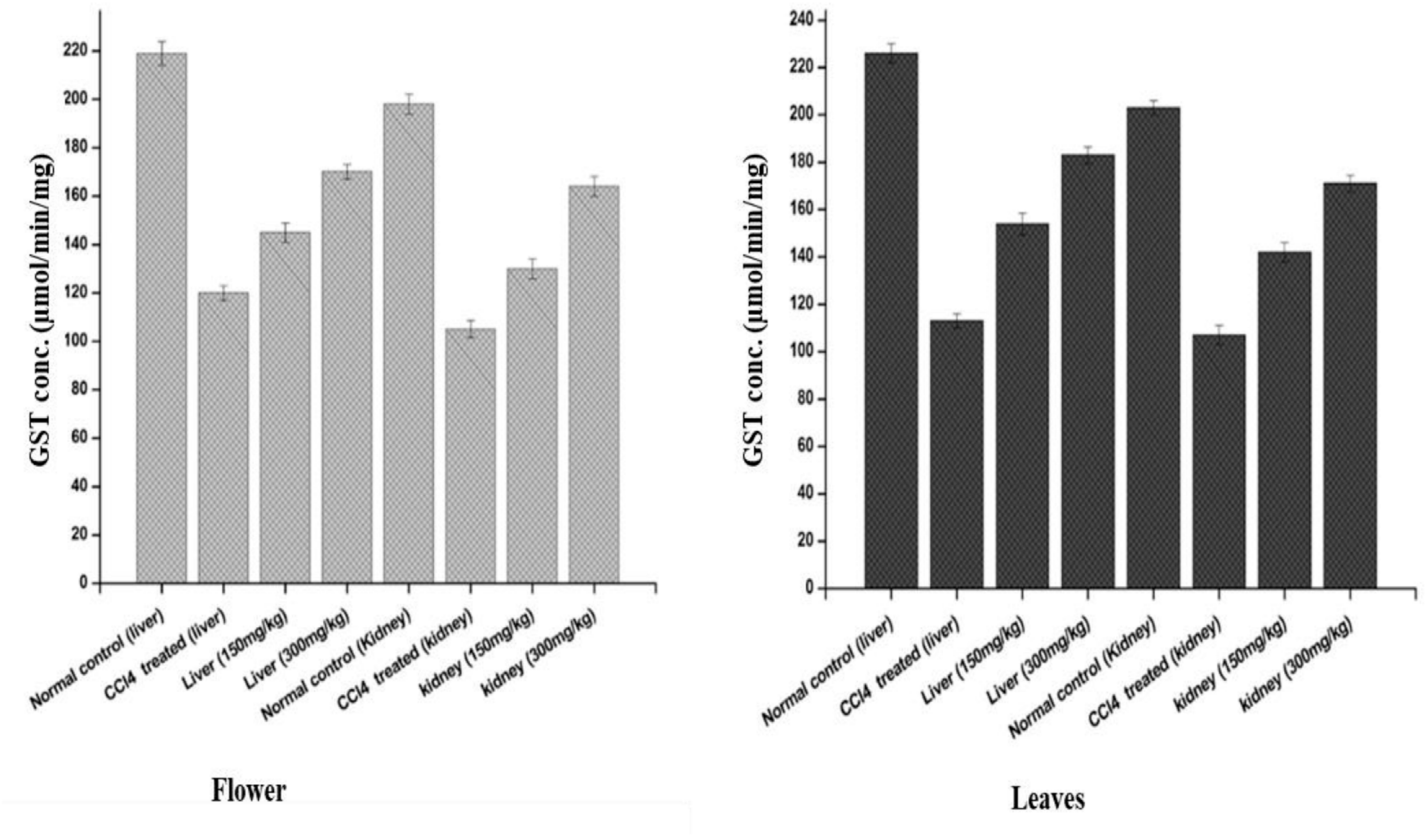
Effects of the plant-DV flower-containing seed and leaf extracts on liver and kidney GST activity *in vivo* of control and experimental groups of rats. Values are expressed as mean ± SD (*n* = 3); *p* < 0.05.

The effects of the plant-DV flower-containing seed extract on *in vivo* liver and kidney GST activities are shown in Figure 6. The GST concentration in both the kidney and liver of normal animals was almost the same (220 µM). The kidneys of those animals which were administered with the 150 ± 2 mg/kg and 300 ± 2 mg/kg flower-containing seed extract showed a significant increase in enzyme activity between the CCl_4_-treated and extract-treated animals. The animals that were given the flower-containing seed extract of 150 ± 2 mg/kg showed an increase in GST from 105 to 130 μmol/min/mg, and the flower-containing seed extract of 300 ± 2 mg/kg showed an increase in GST from 105 to 164 μmol/min/mg. In the livers (Figure 6), the animals treated with the flower-containing seed extract showed a significant increase in enzyme activity between the CCl_4_-treated and extract-treated animals. GST activity in the normal control groups was shown to be 198 μmol/min/mg in the kidney and 219 μmol/min/mg in the liver. The control group comparison showed that the normal control liver yields higher enzymatic activity than the normal control kidney.

An increase in GST activity was detected in the liver and kidney models from the animals administered 150 ± 2 mg/kg and 300 ± 2 mg/kg leaf extracts, as shown in Figure 6. The GST activity increased by 142 and 171 μmol/min/mg for the kidney models administered 150 ± 2 mg/kg and 300 ± 2 mg/kg leaf extracts, respectively. In the liver, as shown in Figure 6, the leaf extract-treated animals showed a substantial increase in enzyme activity at both 150 ± 2 mg/kg and 300 ± 2 mg/kg. GST is markedly shown in the liver but was also observed in other tissues together with the kidney. Liver tissue is the major detoxification site for poisonous substances such as chemical toxins, metal ions, drugs, and cancer-causing metabolites, and detoxification of endogenous noxious molecules. Liver tissue removes endogenous and exogenous toxins using GST in combination with reduced GSH with the electrophile center by the formation of a thioester bond between the substrate and the sulfur atom of GSH (Gulçin et al., 2018).

GST plays an important role in detoxifying and metabolizing many xenobiotic and endogenous compounds (Hattab et al., 2023). Many compounds derived from plants possess antioxidant potential. *In vivo* GST induction is highly beneficial in protecting the cells from electrophilic insult, which might be the cause of several diseases, such as cancer and neurodegenerative diseases, by taking electrons from macromolecules, such as DNA, and proteins (van Haaften et al., 2003).

### 3.4 Assessment of catalase activity

#### 3.4.1 Leaves showed better catalase activity than flower-containing seeds in *in vitro* evaluation

**FIGURE 7:**
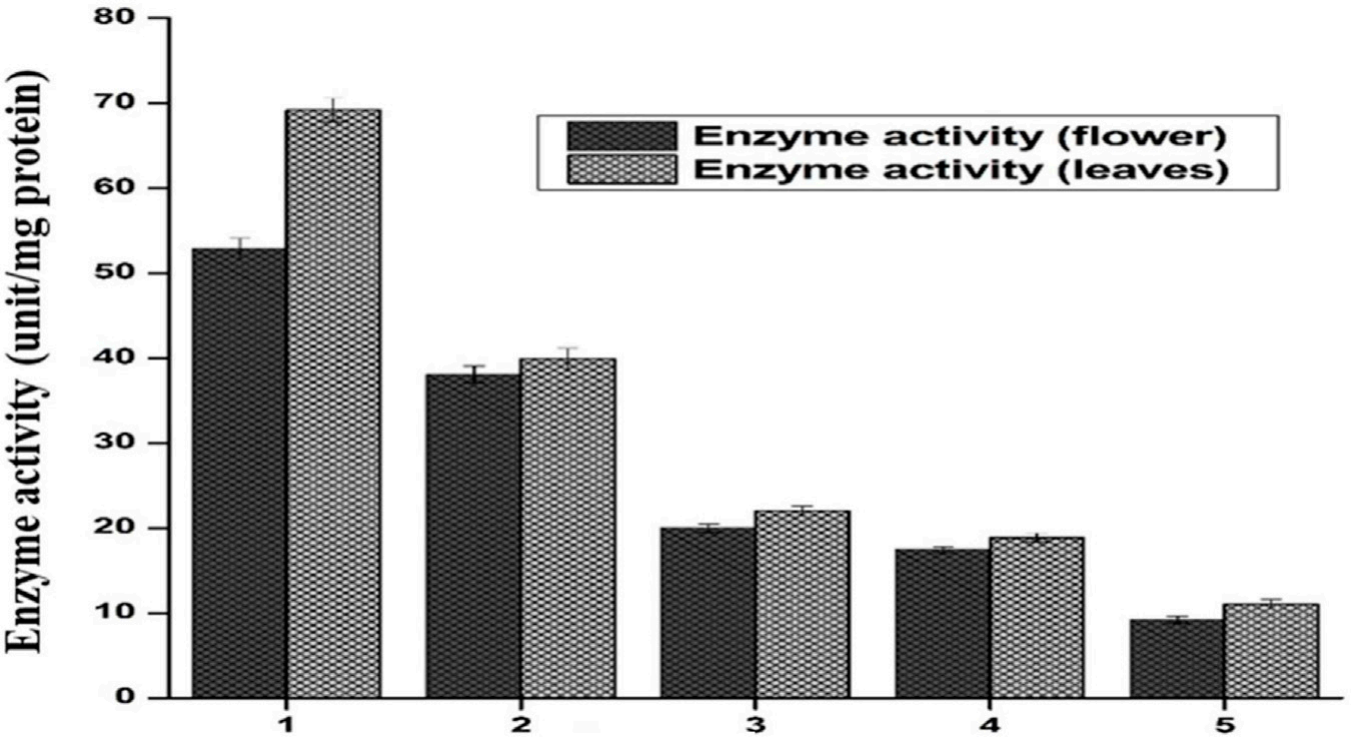
Catalase activity is expressed in units/minute, both the flower and leaf part of plant DV. Values are expressed as mean ± SD (*n* = 3); *p* < 0.05.

The supernatant of flower-containing seeds and the leaves part of plant DV was assessed for the determination of catalase activity. Five serial supernatant dilutions of flower-containing seeds and leaves were prepared by mixing the supernatant with phosphate buffer and observed for CAT activity. These dilutions spectrophotometrically provide altered absorbance in decreasing order with the decreased extract concentration. Figure 7 shows the CAT activity for all five dilutions. The amount of enzyme unit was calculated as the decrease in absorbance at a wavelength of 240 nm by 0.05 units. The activity of CAT in each of the five dilutions was evaluated by the method (Naz et al., 2022). As shown in Figure 7, CAT activity in the flower-containing seed extract was found to be higher in the sample having a maximum supernatant concentration (DV-1) as of 52.88 unit/mg protein. The CAT activity gradually decreased as the supernatant was further diluted with aqueous phosphate buffer. The CAT activity in samples having a minimum supernatant concentration was 9.2 unit/mg protein (DV-5). The respective CAT activity in each dilution (DV-2, DV-3, DV-4, and DV-5) is shown in Figure 7.

CAT activity in the leaf extract was also found to be higher in the sample having a maximum supernatant concentration (DV-1). The maximum supernatant concentration showed a catalytic activity of 69.2 unit/mg protein. The CAT activity gradually decreased as the supernatant was further diluted with aqueous phosphate buffer. The minimum supernatant concentration denoted by DV-5 showed a catalytic activity of 11.04 unit/mg protein. The respective CAT activity in each sample (DV-2, DV-3, DV-4, and DV-5) is shown in Figure 7. In more concentrated samples, there were more CAT enzymes, while in a diluted sample, there may be fewer enzymes available to detoxify the free radicals. CAT catalyzed H_2_O_2_ into H_2_O and O_2_. Due to this conversion, a decrease in absorbance was noted using the UV-Vis spectrophotometer at *λ* = 240 nm. Catalase has been reported to be a common enzyme present in almost all cells exposed to oxygen, and it decomposes hydrogen peroxide into less reactive oxygen and water (Shim et al., 2003; Baker et al., 2023). Previous studies showed a positive correlation with these findings that plant-DV has catalase activity (MacRae and Ferguson, 1985).

#### 3.4.2 *In vivo* evaluation reveals higher catalase activity in leaves than in flower-containing seeds

**FIGURE 8:**
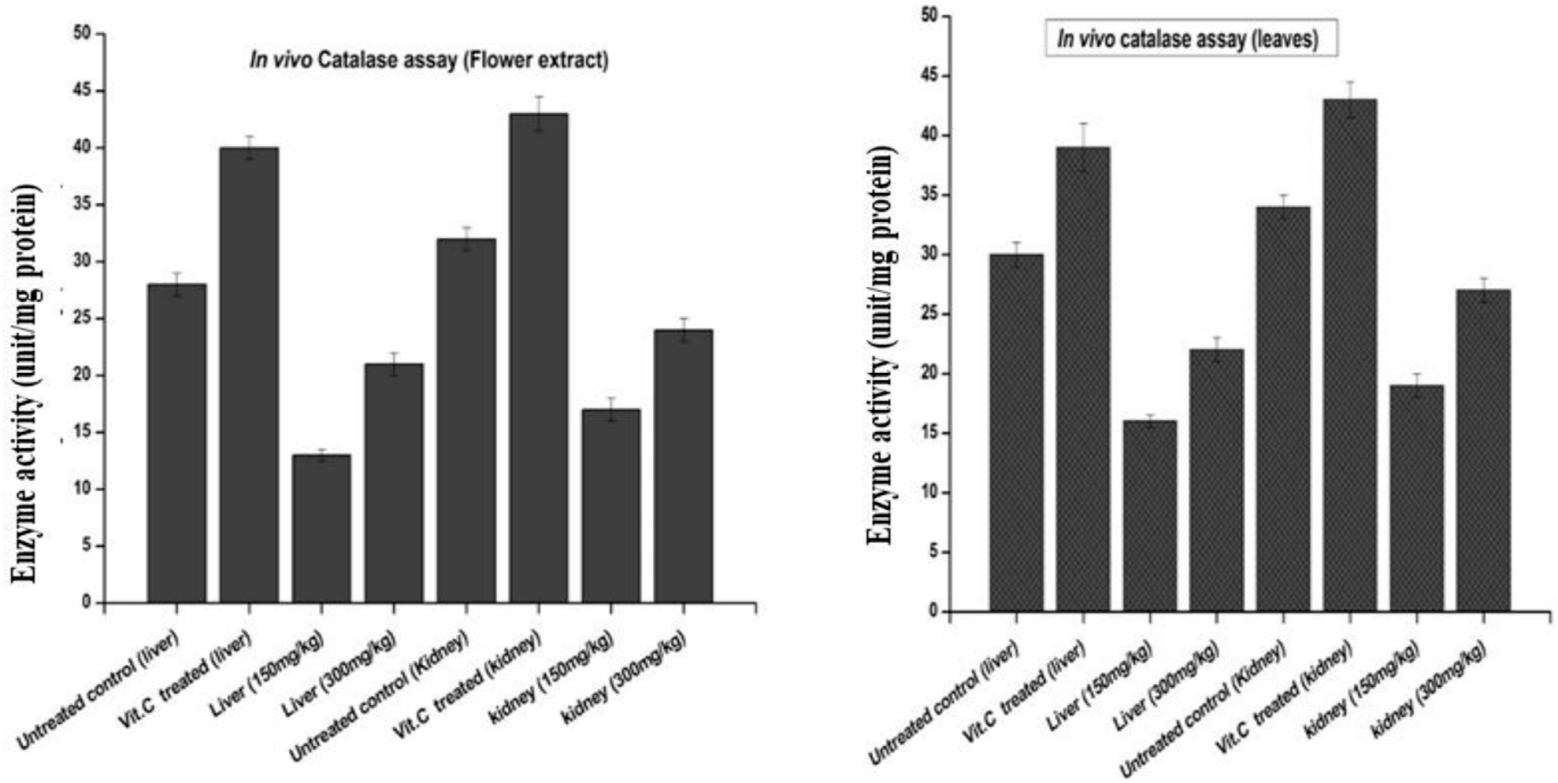
Effects of plant-DV flower-containing seed and leaf extracts on the liver and kidney GST activity *in vivo* of control and experimental groups of rats. Values are expressed as mean ± SD (*n* = 3); *p* < 0.05.

The results of catalase activity, as shown in Figure 8, revealed an increase in liver and kidney tissues for the groups treated with the flower-containing seed extract of 150 ± 2 mg/kg and 300 ± 2 mg/kg with a value of 13 and 17, and 21 and 24 unit/mg protein, respectively, compared to the untreated/negative control group with the value of 28 and 32 unit/mg protein and the vitamin C-treated control group with the value of 40 and 43 unit/mg protein, respectively.

The results of catalase activity also showed an increase in liver and kidney tissue for the groups treated with the leaf extract, 150 ± 2 and 300 ± 2 mg/kg with the value of 16 and 19 and 22 and 27 unit/mg protein, respectively, compared to the untreated/negative control group with the value of 30 and 34 unit/mg protein and the vitamin C-treated control group with the value of 39 and 43 unit/mg protein, respectively.

CAT is an enzymatic antioxidant widely distributed in all animal tissues. It decomposes hydrogen peroxide and protects the tissue from highly reactive hydroxyl radicals. CAT is a part of the antioxidant enzymes that play an indispensable role in the antioxidant protective capacity of biological systems against ROS by regulating the production of free radicals and their metabolites, so the inhibition of these enzymes can cause ROS accumulation and cellular damage (Valenzuela-Cota et al., 2019). Inhibition of this enzyme may enhance sensitivity to free radical-induced cellular damage. Therefore, a reduction in CAT activity may lead to deleterious effects because of superoxide and hydrogen peroxide assimilation.

Briefly, plant DV shows antioxidant potential because they contain a variety of bioactive substances. Antioxidants are one of the vital ingredients of plants (present in different parts) because they are responsible for various normal processes such as adopting environmental stress, being involved in biochemical processes, and growth and development. The leaves and flowers parts of plant DV showed antioxidant potential, which might be due the presence of the same type of active constituents such as flavonoids, polyphenol, and other potent molecules (Malik et al., 2022). Overall, the evaluation of leaves and flowers contributed to health-promoting properties.

## 4 Conclusion

This study reveals the promising antioxidant potential of leaves and seeds of plant DV in *in vitro* and *in vivo* evaluation. *In vitro* evaluation of nascent extracts confirmed concentration-dependent antioxidant potential of both parts having variable results in terms of various antioxidant entities and activities. Oxidatively stressed samples with Ag(I) and Hg(II) showed a concentration-dependent gradual decrease in the antioxidant effect and efficacy of the extracts against metallic oxidation. The strong antioxidant effect of seed and leaf extracts improved the antioxidant capability of the kidneys and liver of treated animals compared to untreated and intoxicated animals with CCl_4_ but less potent than the standard drug, vitamin C. Bioactivity-guided evaluation of seeds and leaves from plant DV will help obtain pure antioxidant compounds and, hence, a way to novel drugs. However, the strong antioxidant potential of the crude extract can be exploited in the treatment and amelioration of various diseases to avoid the loss of micro-ingredients having fortifying, additive, or synergistic correlation.

## Data Availability

The original contributions presented in the study are included in the article/Supplementary Material; further inquiries can be directed to the corresponding authors.

## References

[B1] Alanazi A. Z. Al-Rejaie S. S. Ahmed M. M. Alhazzani K. Alhosaini K. Sobeai H. M. A. (2023). Protective role of Dodonaea viscosa extract against streptozotocin-induced hepatotoxicity and nephrotoxicity in rats. Saudi Pharm. J. 31 (8), 101669. 10.1016/j.jsps.2023.06.002 37576853 PMC10415224

[B2] Al-Snafi A. E. (2017). A review on Dodonaea viscosa: a potential medicinal plant. IOSR J. Pharm. 7 (2), 10–21. 10.9790/3013-0702011021

[B3] Aras A. Korkmaz T. Bayrakdar A. (2023). LC/MS/MS phenolic profile, antioxidant capacity, angiotensin‐converting enzyme (ACE) and glutathione S transferase (GST) inhibitory properties of ultrasound‐assisted propolis extracts. Chem. Biodivers. 20 (4), e202300049. 10.1002/cbdv.202300049 36866854

[B4] Baker A. Lin C.-C. Lett C. Karpinska B. Wright M. H. Foyer C. H. (2023). Catalase: a critical node in the regulation of cell fate. Free Radic. Biol. Med. 199, 56–66. 10.1016/j.freeradbiomed.2023.02.009 36775107

[B5] Benavides M. P. Gallego S. M. Tomaro M. L. (2005). Cadmium toxicity in plants. Braz. J. plant physiology 17 (1), 21–34. 10.1590/s1677-04202005000100003

[B6] Doherty V. Ogunkuade O. Kanife U. (2010). Biomarkers of oxidative stress and heavy metal levels as indicators of environmental pollution in some selected fishes in Lagos, Nigeria. American-Eurasian J. Agric. Environ. Sci. 7 (3), 359–365.

[B7] Dziechciarz P. Strachecka A. Borsuk G. Olszewski K. (2023). Effect of rearing in small-cell combs on activities of catalase and superoxide dismutase and total antioxidant capacity in the hemolymph of *Apis mellifera* workers. Antioxidants 12 (3), 709. 10.3390/antiox12030709 36978956 PMC10044930

[B8] Ellman G. L. (1959). Tissue sulfhydryl groups. Archives Biochem. biophysics 82 (1), 70–77. 10.1016/0003-9861(59)90090-6 13650640

[B9] Farombi E. O. Adelowo O. Ajimoko Y. (2007). Biomarkers of oxidative stress and heavy metal levels as indicators of environmental pollution in African cat fish (*Clarias gariepinus*) from Nigeria Ogun River. Int. J. Environ. Res. Public health 4 (2), 158–165. 10.3390/ijerph2007040011 17617680 PMC3728582

[B10] Gallego S. M. Benavides M. P. Tomaro M. L. (1996). Effect of heavy metal ion excess on sunflower leaves: evidence for involvement of oxidative stress. Plant Sci. 121 (2), 151–159. 10.1016/s0168-9452(96)04528-1

[B11] Getie M. Gebre-Mariam T. Rietz R. Höhne C. Huschka C. Schmidtke M. (2003). Evaluation of the anti-microbial and anti-inflammatory activities of the medicinal plants Dodonaea viscosa, Rumex nervosus and Rumex abyssinicus. Fitoterapia 74 (1-2), 139–143. 10.1016/s0367-326x(02)00315-5 12628410

[B12] Gulçin İ. Taslimi P. Aygün A. Sadeghian N. Bastem E. Kufrevioglu O. I. (2018). Antidiabetic and antiparasitic potentials: inhibition effects of some natural antioxidant compounds on α-glycosidase, α-amylase and human glutathione S-transferase enzymes. Int. J. Biol. Macromol. 119, 741–746. 10.1016/j.ijbiomac.2018.08.001 30076927

[B13] Habig W. H. Pabst M. J. Jakoby W. B. (1974). Glutathione S-transferases. J. Biol. Chem. 249 (22), 7130–7139. 10.1016/s0021-9258(19)42083-8 4436300

[B14] Hattab S. Boughattas I. Cappello T. Zitouni N. Touil G. Romdhani I. (2023). Heavy metal accumulation, biochemical and transcriptomic biomarkers in earthworms *Eisenia andrei* exposed to industrially contaminated soils from south-eastern Tunisia (Gabes Governorate). Sci. Total Environ. 887, 163950. 10.1016/j.scitotenv.2023.163950 37164086

[B15] Herrera-Calderon O. Herrera-Ramírez A. Cardona G. W. Melgar-Merino E. J. Chávez H. Pari-Olarte J. B. (2023). Dodonaea viscosa Jacq. induces cytotoxicity, antiproliferative activity, and cell death in colorectal cancer cells via regulation of caspase 3 and p53. Front. Pharmacol. 14, 1197569. 10.3389/fphar.2023.1197569 37426815 PMC10326442

[B16] Hossain M. A. Piyatida P. da Silva Jat Fujita M. (2012). Molecular mechanism of heavy metal toxicity and tolerance in plants: central role of glutathione in detoxification of reactive oxygen species and methylglyoxal and in heavy metal chelation. J. Bot. 2012, 1–37. 10.1155/2012/872875

[B17] Jayachitra A. Krithiga N. (2012). Study on antioxidant property in selected medicinal plant extract. Int. J. Med. Arom. Plants 2 (3), 495–500.

[B18] Jones D. P. (2002). Redox potential of GSH/GSSG couple: assay and biological significance. Methods Enzym. 348, 93–112. 10.1016/s0076-6879(02)48630-2 11885298

[B19] Jozefczak M. Remans T. Vangronsveld J. Cuypers A. (2012). Glutathione is a key player in metal-induced oxidative stress defenses. Int. J. Mol. Sci. 13 (3), 3145–3175. 10.3390/ijms13033145 22489146 PMC3317707

[B20] Kamran M. Kousar R. Ullah S. Khan S. Umer M. F. Rashid H. U. (2020). Taxonomic distribution of medicinal plants for alzheimer’s disease: a cue to novel drugs. Int. J. Alzheimer’s Dis. 2020, 1–15. 10.1155/2020/7603015

[B21] Khalil N. Sperotto J. Manfron M. (2006). Antiinflammatory activity and acute toxicity of Dodonaea viscosa. Fitoterapia 77 (6), 478–480. 10.1016/j.fitote.2006.06.002 16884859

[B22] MacRae E. Ferguson I. (1985). Changes in catalase activity and hydrogen peroxide concentration in plants in response to low temperature. Physiol. Plant. 65 (1), 51–56. 10.1111/j.1399-3054.1985.tb02358.x

[B23] Malik M. N. Haq I.-u. Fatima H. Ahmad M. Naz I. Mirza B. (2022). Bioprospecting Dodonaea viscosa Jacq.; a traditional medicinal plant for antioxidant, cytotoxic, antidiabetic and antimicrobial potential. Arabian J. Chem. 15 (3), 103688. 10.1016/j.arabjc.2022.103688

[B24] Marvilliers A. Illien B. Gros E. Sorres J. Kashman Y. Thomas H. (2020). Modified clerodanes from the essential oil of dodonea viscosa leaves. Molecules 25 (4), 850. 10.3390/molecules25040850 32075135 PMC7070720

[B25] Moron M. S. Depierre J. W. Mannervik B. (1979). Levels of glutathione, glutathione reductase and glutathione S-transferase activities in rat lung and liver. Biochimica biophysica acta (BBA)-general Subj. 582 (1), 67–78. 10.1016/0304-4165(79)90289-7 760819

[B26] Mostafa A. E. Atef A. Mohammad A.-E. I. Jacob M. Cutler S. J. Ross S. A. (2014). New secondary metabolites from Dodonaea viscosa. Phytochem. Lett. 8, 10–15. 10.1016/j.phytol.2013.12.008

[B27] Muhammad A. Tel-Çayan G. Öztürk M. Duru M. E. Nadeem S. Anis I. (2016). Phytochemicals from Dodonaea viscosa and their antioxidant and anticholinesterase activities with structure–activity relationships. Pharm. Biol. 54 (9), 1649–1655. 10.3109/13880209.2015.1113992 26866457

[B28] Naz H. Akram N. A. Ashraf M. Hefft D. I. Jan B. L. (2022). Leaf extract of neem (Azadirachta indica) alleviates adverse effects of drought in quinoa (Chenopodium quinoa Willd.) plants through alterations in biochemical attributes and antioxidants. Saudi J. Biol. Sci. 29, 1367–1374. 10.1016/j.sjbs.2022.01.038 35280556 PMC8913546

[B29] Prakash J. Gupta S. Kochupillai V. Singh N. Gupta Y. Joshi S. (2001). Chemopreventive activity ofWithania somnifera in experimentally induced fibrosarcoma tumours in Swiss albino mice. Phytotherapy Res. Int. J. Devoted Pharmacol. Toxicol. Eval. Nat. Prod. Deriv. 15 (3), 240–244. 10.1002/ptr.779 11351360

[B30] Ruch R. J. Cheng S.-j. Klaunig J. E. (1989). Prevention of cytotoxicity and inhibition of intercellular communication by antioxidant catechins isolated from Chinese green tea. Carcinogenesis 10 (6), 1003–1008. 10.1093/carcin/10.6.1003 2470525

[B100] Samavati V. Manoochehrizade A. (2013). Dodonaea viscosa var. angustifolia leaf: new source of polysaccharide and its anti-oxidant activity. Carbohydr. Polym. 98 (1), 199–207. 23987336 10.1016/j.carbpol.2013.05.083

[B31] Shalaby N. Abd-Alla H. Hamed M. Al-Ghamdi S. Jambi S. (2012). Flavones composition and therapeutic potential of Dodonaea viscosa against liver fibrosis. Int. J. Phytomedicine 4 (1), 27.

[B32] Shim I.-S. Momose Y. Yamamoto A. Kim D.-W. Usui K. (2003). Inhibition of catalase activity by oxidative stress and its relationship to salicylic acid accumulation in plants. Plant Growth Regul. 39 (3), 285–292. 10.1023/a:1022861312375

[B33] Siddiqui N. A. Almarfadi O. M. Shahat A. A. Alqahtani A. S. El Gamal A. A. Raish M. (2023). Isolation of new compound and neuroprotective studies from Dodonaea viscosa. J. King Saud University-Science 35 (5), 102704. 10.1016/j.jksus.2023.102704

[B34] Sidra J. Hanif M. Khan M. Qadri R. (2014). Natural products sources and their active compounds on disease prevention: a review. Int. J. Chem. Biochem. Sci. 6, 76–83.

[B35] Simpson B. S. Claudie D. J. Smith N. M. Gerber J. P. McKinnon R. A. Semple S. J. (2011). Flavonoids from the leaves and stems of Dodonaea polyandra: a Northern Kaanju medicinal plant. Phytochemistry 72 (14-15), 1883–1888. 10.1016/j.phytochem.2011.05.006 21641623

[B36] Skenderidis P. Kerasioti E. Karkanta E. Stagos D. Kouretas D. Petrotos K. (2018). Assessment of the antioxidant and antimutagenic activity of extracts from goji berry of Greek cultivation. Toxicol. Rep. 5, 251–257. 10.1016/j.toxrep.2018.02.001 29854596 PMC5977381

[B37] Srnovršnik T. Virant-Klun I. Pinter B. (2023). Heavy metals and essential elements in association with oxidative stress in women with polycystic ovary syndrome—a systematic review. Antioxidants 12 (7), 1398. 10.3390/antiox12071398 37507937 PMC10376316

[B38] Subramanian S. Duraipandian C. Alsayari A. Ramachawolran G. Wong L. S. Sekar M. (2023). Wound healing properties of a new formulated flavonoid-rich fraction from Dodonaea viscosa Jacq. leaves extract. Front. Pharmacol. 14, 1096905. 10.3389/fphar.2023.1096905 36817128 PMC9932054

[B39] Valenzuela-Cota D. F. Buitimea-Cantúa G. V. Plascencia-Jatomea M. Cinco-Moroyoqui F. J. Martínez-Higuera A. A. Rosas-Burgos E. C. (2019). Inhibition of the antioxidant activity of catalase and superoxide dismutase from Fusarium verticillioides exposed to a Jacquinia macrocarpa antifungal fraction. J. Environ. Sci. Health, Part B 54 (8), 647–654. 10.1080/03601234.2019.1622978 31146638

[B40] van Haaften R. I. Haenen G. R. van Bladeren P. J. Bogaards J. J. Evelo C. T. Bast A. (2003). Inhibition of various glutathione S-transferase isoenzymes by RRR-α-tocopherol. Toxicol. vitro 17 (3), 245–251. 10.1016/s0887-2333(03)00038-9 12781202

[B41] Zhu Y. Wang K. Jia X. Fu C. Yu H. Wang Y. (2023). Antioxidant peptides, the guardian of life from oxidative stress. Med. Res. Rev. 10.1002/med.21986 37621230

